# Women Do Not Utilise Family Planning According to Their Needs in Southern Malawi: A Cross-Sectional Survey

**DOI:** 10.3390/ijerph18084072

**Published:** 2021-04-13

**Authors:** Maria Lisa Odland, Oda Vallner, Marlen Toch-Marquardt, Elisabeth Darj

**Affiliations:** 1 Institute of Applied Health Research, Birmingham University, Birmingham B15 2TT, UK; 2 Department of Public Health and Nursing, NTNU, Norwegian University of Science and Technology, 7491 Trondheim, Norway; oda.vallner@gmail.com (O.V.); marlen.toch@ntnu.no (M.T.-M.); elisabeth.darj@ntnu.no (E.D.); 3 Department of Obstetrics and Gynaecology, St Olavs Hospital, 7030 Trondheim, Norway

**Keywords:** family planning, unmet need, contraception, Malawi

## Abstract

Malawi is a low-income country with a high maternal mortality rate. This study aimed to investigate the use of contraception and factors associated with unmet need of family planning among fertile women in selected health facilities in southern Malawi. A cross-sectional study design was employed using a validated questionnaire to investigate the unmet need. A total of 419 pregnant women, who attended antenatal clinics at a central hospital and two district hospitals, voluntarily participated in the study. Logistic regression analysis was used to identify possible factors associated with unmet needs. Amongst the participants, 15.1% reported unmet need, 27.0% had never used a contraceptive method, and 27.2% had an unwanted pregnancy. Being married, 20–24 years of age, living in a rural area, and high parity were protective factors against having unmet need regarding family planning. Malawi, a country with a young population and a high fertility rate, has a high level of unmet family planning need. Barriers and facilitators need to be identified and addressed at different levels by the health care system, society, and the government of Malawi.

## 1. Introduction

Maternal mortality is still a big problem globally, but especially in low-income countries. Malawi is a low-income country with a population of 18 million [1]. The country has a fertility rate of 4.4 [2] and a maternal mortality ratio (MMR) of 439 per 100,000 women, which is among the highest in the world [2]. About 6–30% of all maternal deaths in Malawi are estimated to be caused by unsafe abortions [3], and an estimated 54% of pregnancies in Malawi were unintended in 2013 [4]. According to the restrictive abortion law, termination of pregnancy is only legal in order to save a pregnant woman’s life [5]. Still, 140,000 induced abortions were estimated in the country in 2015, and considering the illegality of abortions, the majority were most likely unsafe [6]. Legalizing abortions has led to a decrease in maternal mortality in other countries [7], and liberalising the abortion law in Malawi has been discussed for years, but so far no change has happened. Whilst we are waiting for the abortion law to change, other efforts to reduce maternal mortality need to be undertaken. In a report by the Guttmacher Institute from 2014, it was estimated that if all unmet family planning needs were met for women in Malawi, unintended births and unsafe abortions would drop by 87% and maternal mortality would decline by more than 40% [4].

One way to reduce the MMR could by improving access to family planning. A systematic review regarding the usage of contraceptives in Sub-Saharan Africa found major barriers preventing the uptake of family planning services, such as cultural and societal pressure on women, socioeconomic status, financial barriers, and lack of access to services [8]. An Ugandan study points to barriers leading to reduced access to contraceptives as misconceptions, gender power relations, socio-cultural expectations, judgmental health services, lack of privacy, and confidentiality as public health concerns that are possibly associated with mortality among women attributed to low contraceptive use [9]. Consequently, identifying the barriers and facilitators to family planning is important. An unmet need of family planning (unmet need) is defined as the proportion of women who want to stop or delay childbearing but who are not using any method of contraception [10]. In the Demographic and Health Survey (DHS) from 2015–2016, the unmet need was estimated to be 18.7% among married women and 39.8% for sexually active unmarried women [2]. An assessment by the WHO, Ipas, and the Malawian Ministry of Health in 2011 summarized four key recommendations to reduce the MMR; strengthening the national family planning programme; addressing the sexual and reproductive health needs of young Malawians; reviewing restrictive abortion laws; and strengthening post abortion care services [3]. Thus, family planning is recognized as an important factor to achieve a reduction in unwanted pregnancies, early motherhood, and unsafe abortions (consequently reducing maternal mortality). In order to increase access to family planning and reduce unmet need, barriers in access to family planning need to be determined before they can be addressed. This study aimed to investigate the prevalence of the unmet need among a selected group of fertile women in southern Malawi and possible factors associated with unmet need within this group.

## 2. Materials and Methods

### 2.1. Study Design, Setting, and Participants

We used a cross-sectional survey design to investigate the unmet need of contraceptives among women in Malawi. The study was conducted at the Queen Elizabeth Central Hospital (QECH) in Blantyre, and at the district hospitals of Chikwawa and Chiradzulu. We aimed to include as many women of reproductive age as possible during the study period of five weeks. A convenience sampling method was used to target pregnant women aged 16 to 49 years attending the antenatal clinic (ANC) at the selected health facilities. All women attending the ANCs were invited for voluntary participation.

A validated, 18 question questionnaire, based on “Questions and Filters for unmet need Definition” from the WHO, was used in the data collection [11]. We added 11 questions to describe sociodemographic characteristics. The questionnaire was available in English and a translated version in Chichewa. The nurse in charge informed the women about the study in Chichewa and of their right to decline to participate without this affecting their treatment at the antenatal clinic. The nurses who administrated the questionnaires were also available to read the questionnaire for the participants if necessary. There were also posters with information about the study at each ANC. The women who agreed to participate received a questionnaire and could answer it in private whilst waiting for their antenatal check-ups. Informed consent forms were signed by the participants, or with thumb print if illiterate. The participants did not receive any compensation for completing the questionnaire, but were offered refreshments whilst completing the form.

### 2.2. Description of Variables

The dependent variable unmet need was constructed from three different parts: (1) women who did not want to get pregnant and did not use anything to avoid pregnancy; (2) women who wanted to have another child later, but would not use anything to space pregnancies; and (3) women who did not want any more children and would not use anything to limit childbearing.

Age was categorized into five-year groups (16–19, 20–24, 25–29, 30–34, and ≥35), area of living was divided into urban (Blantyre) or rural (Chikwawa or Chiradzulu), achieved level of education in three categories (low = primary school, middle = secondary school, and high = higher education), and marital status was married or non-married (single, widowed, separated, or cohabiting).

### 2.3. Analyses

Data from the questionnaires were entered manually into a statistical software package SPSS 20.0. Descriptive statistics were computed for all variables used in the analysis. Logistic regression analysis was used to calculate odds ratios (ORs) and 95% confidence intervals (CIs) to identify possible factors associated with unmet need. We calculated one model including area of living (Model I) and a second model adjusting for possible confounding factors (Model II).

## 3. Results

### 3.1. Descriptive Results

The number of women visiting the ANCs at the approached three health facilities amounted on average to 420 every week, and the response rate was 40%. During four weeks in October–November 2017, 419 women were included in the study. Most women were aged 25–29 years and almost everyone was married (92.1%). The average number of children was 1.5 child (range 0–8) per woman and 33.7% were pregnant with their first child (Table 1).

The total unmet need in our study was 15.1% and the age group 20–24 years had the lowest odds of unmet need. Women attending the antenatal clinic at the QECH in Blantyre had a higher unmet need (20.1%). These women were also older and had higher educational levels. In the district areas the unmet need was lower; Chiradzulu (15.1%) and Chikwawa (7.3%) (Table 1). 

As indicated in Table 2, 27.0% of the included women had never used contraceptives, and 85% of these women wished that they had been able access to contraception. The women who used contraceptives normally received them from a hospital or from clinics in rural areas. The most frequently used contraceptives were injections (58.2%) and implants (26.5%). Few women had access to IUDs (3.6%), or emergency contraception (2.0%) after unprotected intercourse. A substantial proportion of respondents (45.0%) reported side effects of their contraceptives, with bleeding disturbance being the most common side effect (43.5%) (Table 2).

Close to a third of the women (27.2%) did not want or did not plan their current pregnancy. However, 65.0% wanted another child, but the current pregnancy was mistimed, and 78.4% wished to be able to space the interval between pregnancies by at least two years. The most frequent choices the women considered for spacing pregnancies were injections (46.4%) or implants (26.3%) (Table 3).

Only sixteen women did not want to use contraceptives for spacing or preventing a pregnancy. This was due to various reasons, such as, the husband opposed the use of contraceptives, experiences of side effects, inconvenience, lack of access, or the belief that the occurrence of pregnancy is decided by God.

### 3.2. Logistic Regression Analysis 

Women living in the district of Chikwawa had a statistically significantly lower OR, 0.30 (95% CI 0.13–0.68), of unmet need compared to women living in Blantyre (Table 4). Moreover, women in the age group 20–24 had the lowest OR, 0.29 (95% CI 0.12–0.71), for unmet need compared to other age groups. Education was not associated with unmet need. Married women had a lower OR of unmet need than unmarried women, and the odds of having unmet need decreased with increasing parity (Table 4).

## 4. Discussion

In this study we found that three quarters of the included Malawian women have used contraceptives, but 15.1% reported a current unmet need. Almost a third of the participants had an unwanted pregnancy. 

Malawi has a young population, with an average age of 17.2 years in 2015 [12]. The median age at marriage is 17.8 years in the southern parts of Malawi and women have high fertility [2]. The annual population growth in Malawi was 2.6% in 2018 [1,2]. However, worldwide the estimation of population growth is currently decreasing, and the overall number of women who have access and are using contraceptives is growing [13]. In a Guttmacher report from 2016, in which DHS surveys were conducted in 52 developing countries where, between 8% and 38% of married women aged 15–49 years reported an unmet need of contraception [14]. They wanted to avoid a pregnancy but did not use any method to prevent a new pregnancy [14]. Our estimate of unmet need is somewhat lower than that reported by previous studies from Malawi [2,15]. In the Malawian DHS the unmet need was 20%, and 54% of pregnancies were estimated to be unintended among married women [2]. This difference in results may indicate that the situation in Malawi is improving and more women are having access to family planning. We would have expected a higher level of unmet need in the Malawi female population, since we included also unmarried women, which is a factor associated with an unmet need [2]. However, another factor that is important to consider is that we asked women at the ANCs. While some of these women had unwanted pregnancies they cannot be considered a representative sample of the fertile population, which could have influenced the results. To include women treated for incomplete abortions or a random sample of the population may have revealed higher unmet need. While there may be a trend towards improvement in Malawi, the unmet need we found in our study was still high and needs to be addressed further.

Unmet need tends to be higher among women with a lower educational level, living in rural areas, or those who have a low income, compared to women who are more educated and living in urban areas [14]. However, this partly contradicts our study where we found that women living in the urban area were more likely to have unmet need. Women in Blantyre have better access to schools and universities, and they ought to have better access to contraceptives. In Lao DPR adolescents living in rural districts had lower sexual and reproductive health knowledge compared to urban district residents [16]. One explanation of our findings might be that the urban women have more knowledge regarding family planning, but they do not perceive that they have access to the contraceptives they need. As expected, being younger, unmarried, and having lower education were factors associated with unmet need in our study. Unmet need may lead to teenage pregnancies, girls dropping out of school, early marriages, and loss of opportunities [17].

The age group 20–24 years had the lowest odds of unmet need, and reasons for this may be that women in this age group often are newly married and want to start families. The literacy rate among women in Malawi is about 55% [18]. The young generation with current access to schools, and thus better education, may become more informed about contraception and methods of family planning. The Ministry of Health in 2001 stated an aim to address the sexual and reproductive health needs of young Malawians [2,4]. Simultaneously, we found that the youngest (16–20 years) and the highest age groups (≥35) had higher odds of unmet need. The teenagers living in rural areas also often have lower educational levels, as presented in the Malawi DHS [2].

Other studies have named further reasons for the high risk of unmet need among unmarried women in addition to age and education such as infrequent sex, and the shame associated with asking for, or buying, contraceptives whilst not married [14]. A qualitative study from Kenya showed that women were scared of being seen as promiscuous when using contraceptives [19].

Unplanned or unwanted pregnancies occur due to a number of reasons, such as lack of knowledge, accessibility, availability, affordability, contraceptive failure, and poverty [20]. The challenges that women face to access safe childbirth, also known as the “Three Delay Model” by Thaddeus and Maine, in seeking, reaching, and receiving care, can also be applied for women’s access to safe contraception [21]. A woman has to have the knowledge of why, what, and where contraceptives are available. Additionally, she needs money and transportation to such a facility, and to get access to available and affordable contraceptives with few side effects. To use prescribed modern contraceptives, women need financial assets and require medical supervision [22]. In countries with easy access to contraceptives the number of abortions is generally low [23]. In Malawi, where abortion laws are very restrictive, the amount of incomplete abortions is high, probably as a consequence of unsafe and dangerous abortions due to unwanted pregnancies. Hence, the lack of access to safe family planning methods leads to unwanted pregnancies, unsafe abortions, and maternal deaths, is one of the reasons Malawi has one of the highest MMRs in the world [3,24].

Six out of ten women in our study reported that they had used injectable contraceptives, but still there were many barriers in access to family planning. Some women also revealed that their partners opposed the use of contraceptives. How males influence the use of contraceptives has been described in other studies [19,25]. Male opposition was attributed to limited understanding and male dominance in a South African study [26]. Men are often seen as the decision makers in families and influence women’s choice of using or not using contraceptives for spacing pregnancies or stopping childbearing [19,26]. Hence, men play an important role when considering unmet need, and men’s attitudes and practices need to be considered in future interventions. A Kenyan study showed that barriers to the use of contraceptives were associated with misconceptions of promiscuity and fear of side effects or infertility [19]. Almost half of the women in our study who did not use any contraceptives reported side-effects and that contraceptives interfere with bodily processes. Women who used injections and implants reported irregular menstruation, which is a well-known and common side-effect [27]. Additionally, the injection should be administered every three months, which could present a barrier to uptake. A study from Kenya described that women had good knowledge of modern contraceptives, however, this was not necessarily translated into use [19]. Thus, information and counselling are important in the effort to reduce the unmet need and to improve the uptake of more effective methods of contraception, with the ultimate aim to improve reproductive health and maternal mortality [20].

The unmet need and the high fertility rate, together with the identified barriers, need to be addressed at different levels in society. One way is to adhere and implement the recommendations from the Ministry of Health. The aim is to strengthen the family planning programme through education and counselling of sexual and reproductive health, reducing the unmet need of young Malawians, and strengthening the important post abortion care services. It will also be critical to review the country’s restrictive abortion laws, a process already ongoing in other African countries [28,29].

We collected data from more than 400 women in a short period, from three hospitals of various sizes, and from urban and rural areas. Additionally, we included unmarried women in contrast to the other reports [2]. There may be some selection bias, as women who agreed to participate are likely to understand and speak English and have a higher level of education. In order to reduce selection bias, we offered a research assistant to read the questionnaire in Chichewa out loud and tick the boxes according to the woman’s answers. In the validated WHO questionnaire, no questions were included regarding the ability to cover the cost of contraceptives, and it would have been interesting to explore this as a potential barrier. The descriptive results from our study cannot be generalised to all fertile women in Malawi. To access a group of women at childbearing age, we chose to approach women who made their regular antenatal check-up at three ANCs. We are aware that we did not reach a representative population of all fertile women, including those who are not pregnant, those who are pregnant but not attending any antenatal care, and women admitted to hospitals due to on-going complications of miscarriages or abortions. Including women with on-going incomplete abortions could have given a more complete picture of the unmet need in Malawi. However, we found it unethical to approach women with on-going abortions with questions regarding their unmet need.

We did not include men in our survey, as it was conducted at an antenatal clinic where men are not usually present, and we consider this as a limitation. Men play an important role and may influence women’s choice of using contraceptives or not. The International Conference on Reproductive Health, Mumbai (India), arranged by UNFPA/WHO/World Bank, stated already in 1998 that men and women shall have “the same rights to decide freely and responsibly on the number and spacing of their children, and to have access to the information, education and means to enable them to exercise these rights” [30].

## 5. Conclusions

Our results show that there is still high unmet need among women at reproductive age in Malawi. Unmet need was more pronounced among teenagers (16–20 years) and older women (≥35 years), unmarried women, and women living in urban areas. The high fertility rate in combination with unmet need should be addressed by Malawian politicians and policymakers in order to improve women’s lives and empower women to make their own decisions about their future. Reducing unmet need will ultimately reduce unsafe abortions and subsequently maternal mortality, as per the requirements of the UN Sustainable Developing Goals for all countries.

## Figures and Tables

**Table 1:** Characteristics of pregnant women (*n* = 419) attending antenatal clinics at three hospitals in southern Malawi.

	Total		Living Area ^4^		
			Blantyre	Chikwawa	Chiradzulu
			*n* = 144	*n* = 109	*n* = 157
	*n*	%	%	%	%
Unmet Need ^1^					
Yes	62	15.1	20.1	7.3	15.1
No	348	84.9	79.9	92.7	84.9
Age ^2^					
16–19	63	15	4.9	18.3	22.9
20–24	108	25.8	13.3	31.2	33.8
25–29	110	26.3	33.6	22.9	22.9
30–34	87	20.8	37.1	12.8	11.5
35+	49	11.7	11.2	14.7	8.9
Education					
Low	156	37.2	7.6	60.6	49
Middle	187	44.6	46.5	35.8	48.4
High	76	18.1	45.8	3.7	2.5
Marital status					
Married	386	92.1	93.8	92.7	90.4
Unmarried	33	7.9	6.3	7.3	9.6
Parity ^3^					
0	141	33.7	25.0	39.4	33.9
1	94	22.4	23.6	16.5	22.2
2	99	23.6	34.0	16.5	23.7
3+	82	19.6	17.4	27.5	20.2

^1^ 9 cases missing, ^2^ 2 cases missing, ^3^ 3 cases missing, ^4^ 10 cases missing.

**Table 2:** Contraceptive use among pregnant women (*n* = 419) at three hospitals in southern Malawi.

Contraceptive Use	(*n*)	(%)
Use of any method	306	73.0
Never used	113	27.0
Wish for use, if not using	96	85
Modern methods		
Injection	178	58.2
Implant	81	26.5
Pill	52	17.0
Male condom	51	17.0
IUD	11	3.6
ECP	6	2.0
Other	3	1
Traditional		
Rhythm and withdrawal	21	6.9
Side Effects	138	45.0
Bleeding disturbance	60	43.5
Loss of period	8	5.8
Long-lasting effect	3	2.2
Gastrointestinal	18	13.0
Headache	11	8.0
Backache	7	5.1
General pain	7	5.1
Heart palpitations	5	3.6
Hypertension	5	3.6
Other ^1^	17	12.3
Place of receipt		
Hospital	107	35.0
Clinic	23	7.5
Private clinic	16	5.0
Private hospital	2	0.7
Health centre	7	2.0
CHW	3	1.0
Family members	1	0.3
Pharmacy	1	0.3
Shop	7	2.0

IUD = intrauterine device, CHW = community health workers, ECP = emergency contraceptive pills. ^1^ Other includes: dizziness, dyspareunia, loss of libido, tiredness, increased appetite, dysmenorrhea, vaginismus, increased vaginal discharge, hormonal change, mental change, and oedemas.

**Table 3:** Family planning among pregnant women *(n* = 419) at three hospitals in southern Malawi.

Family Planning	Total (*n*)	Total (%)
Wanted pregnancy	304	72.6
Unwanted pregnancy	114	27.2
Mistimed	55	48.0
Wanted to stop childbearing	22	19.0
Desire for another child		
Want more children	276	65.0
Not sure	43	10.3
When		
Within two years	8	2.5
Wait at least two years	250	78.4
Don’t know when	49	15.4
Use of any method to space pregnancy (yes and maybe)	297	90.0
Rhythm and withdrawal	10	3.4
Condom	15	5.1
Pill	14	4.7
Implant	78	26.3
IUD	15	5.1
Injection	138	46.5
Don’t know	26	8.8
Other ^1^	1	0.3
No desire for more children	98	23.4
Any method to stop childbearing (yes and maybe)	85	86.7
Rhythm and withdrawal	1	1.2
Condom	3	3.5
Pill	1	1.2
Implant	11	12.9
IUD	11	12.9
Vaginal rings	4	4.7
Injection	12	14.1
Sterilization (bilateral tubal ligation)	40	47.1
Don’t know	3	3.5
Other	2	2.4

^1^ Includes emergency contraceptive pill.

**Table 4:** Logistic regression analyses for the unmet need of family planning in three hospitals in southern Malawi.

	Model I		Model II ^2^	
	OR	CI (95%)	OR	CI (95%)
Area				
Blantyre (RG) ^1^	1		1	
Chikwawa	0.30	(0.13–0.68)	0.22	(0.08–0.65)
Chiradzulu	0.72	(0.40–1.28)	0.54	(0.23–1.23)
Age				
16–19 (RG)			1	
20–24			0.29	(0.12–0.71)
25–29			0.41	(0.14–1.19)
30–34			1.05	(0.29–3.73)
≥35			2.61	(0.56–12.20)
Education				
High (RG)			1	
Medium			1.27	(0.41–3.94)
Low			1.28	(0.55–3.01)
Marital Status				
Not married (RG)			1	
Married			0.43	(0.18–1.04)
Parity				
0 births (RG)			1	
1			0.32	(0.13–0.79)
2			0.23	(0.08–0.63)
3≤			0.05	(0.01–0.23)
−2 loglikelihood	351.772		303.939 ^2^	

^1^ RG = reference group, OR = odds ratio, CI = confidence interval. ^2^ Adjusted for possible influencing factors (age, parity, education, and marriage).

## Data Availability

The data is not publicly available, but can be made available by request.
